# Revascularization of the necrotic femoral head after traumatic open anterior hip dislocation in a child: a case report

**DOI:** 10.1186/s13256-019-2192-7

**Published:** 2019-08-16

**Authors:** Kenta Momii, Satoshi Hamai, Goro Motomura, Kensuke Kubota, Masato Kiyohara, Takuaki Yamamoto, Yasuharu Nakashima

**Affiliations:** 1 Department of Orthopaedic Surgery, Faculty of Medical Sciences, Kyushu University, 3-1-1 Maidashi, Higashi-ku, Fukuoka, 812-8582 Japan; 2 Emergency and Critical Care center, Kyushu University, 3-1-1 Maidashi, Higashi-ku, Fukuoka, 812-8582 Japan; 3 Department of Orthopaedic Surgery, Faculty of Medicine, Fukuoka University, 7-45-1 Nanakuma, Jonan-ku, Fukuoka, 814-0180 Japan

**Keywords:** Revascularization, Ischemic osteonecrosis, Femoral head, Traumatic hip dislocation

## Abstract

**Introduction:**

Avascular necrosis of the femoral capital epiphysis is the most serious complication after traumatic dislocation of the hip in children. This case report discusses the localization and revascularization of the necrotic femoral head following rarely experienced traumatic open anterior hip dislocation in children.

**Case presentation:**

Our patient was an 11-year-old Japanese boy who had open anterior hip dislocation sustained in a traffic accident. Reduction of the hip joint was performed in an emergency operation, and he was evaluated using serial gadolinium-enhanced magnetic resonance imaging. T1-weighted magnetic resonance images showed two bands with low signal intensity in the femoral capital epiphysis on coronal and oblique axial planes, indicating the existence of avascular osteonecrosis of the femoral head. We observed gadolinium enhancement in the central region of the epiphysis, where the area between the two bands with low signal intensity was located. Serial assessment with enhanced magnetic resonance images during a non-weight-bearing period of 1.5 years after injury showed revascularization starting from the central region and converging toward the peripheral region. Although the patient had leg-length discrepancy due to the early epiphyseal closure, non-weight-bearing treatment for the avascular osteonecrosis of the femoral head achieved a favorable outcome without any hip joint dysfunction, pain, or sign of secondary osteoarthritic change within 4.5 years after injury.

**Conclusion:**

We confirmed the revascularization process of the necrotic lesion in the femoral capital epiphysis in an 11-year-old boy using serial gadolinium-enhanced magnetic resonance imaging. Conservative non-weight-bearing treatment achieved a favorable outcome.

## Introduction

Avascular necrosis of the femoral capital epiphysis is the most serious and feared complication after traumatic dislocation of the hip in children; treatment of the severely collapsed femoral head is challenging because it is often unsalvageable [1–10]. Femoral head collapse causes pain and juvenile end-stage osteoarthritis, which leads to serious hip dysfunction. Therefore, postreduction treatment for ischemic necrosis plays a key role in preventing or minimizing development of femoral head collapse.

Herrera-Soto *et al.* [11] reported that children younger than age 12 years who develop osteonecrosis after traumatic hip dislocation will develop femoral head changes such as those seen with Legg-Calvé-Perthes disease [12, 13]. Kim *et al*. [14, 15] recently examined immature piglet models of ischemic osteonecrosis involving vascular disruption; furthermore, they evaluated the revascularization process in patients with Legg-Calvé-Perthes disease using gadolinium-enhanced magnetic resonance imaging (MRI). They concluded that local non-weight-bearing decreased the femoral head deformity and increased revascularization and resorption of the infarcted epiphysis. However, to the best of our knowledge, no previous report has demonstrated evaluations of the necrotic lesion and/or revascularization response following rarely experienced traumatic hip dislocation in children. We present a case of an 11-year-old boy with avascular necrosis of the femoral head and improvement in blood flow during non-weight-bearing according to serial gadolinium-enhanced MRI after traumatic open anterior hip dislocation.

## Case presentation

**Fig. 1:**
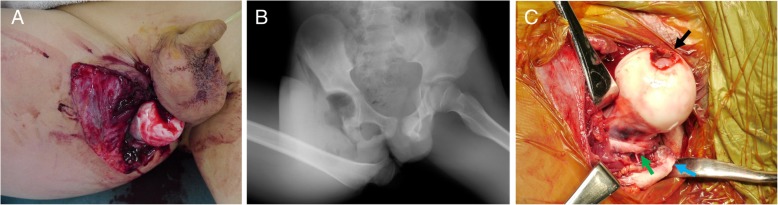
**a** Clinical photograph from the front view showing the femoral head protruding from behind the right adductor muscles. **b** Anteroposterior radiograph showing an anterior inferior dislocation of the right hip joint. **c** Clinical photograph during surgery from the front view showing the posterior aspect of proximal femur showing injuries of ligamentum teres (*black arrow*), gluteus medius, gluteus minimus, iliopsoas, quadratus femoris, short external rotators, and capsule (*green arrow*) with avulsion fractures of the greater (*blue arrow*) and lesser trochanters

Our patient was an 11-year-old Japanese boy who was hit by an automobile while walking. He was admitted to our hospital by a rescue team approximately 30 minutes after injury. Physical examination revealed exposure of the right femoral head from the inside of the thigh (Fig. 1a), presence of ipsilateral dorsalis artery pulsation, and no signs of neurological impairment. Initial radiographs revealed anterior dislocation of the right hip (Fig. 1b).

**Fig. 2:**
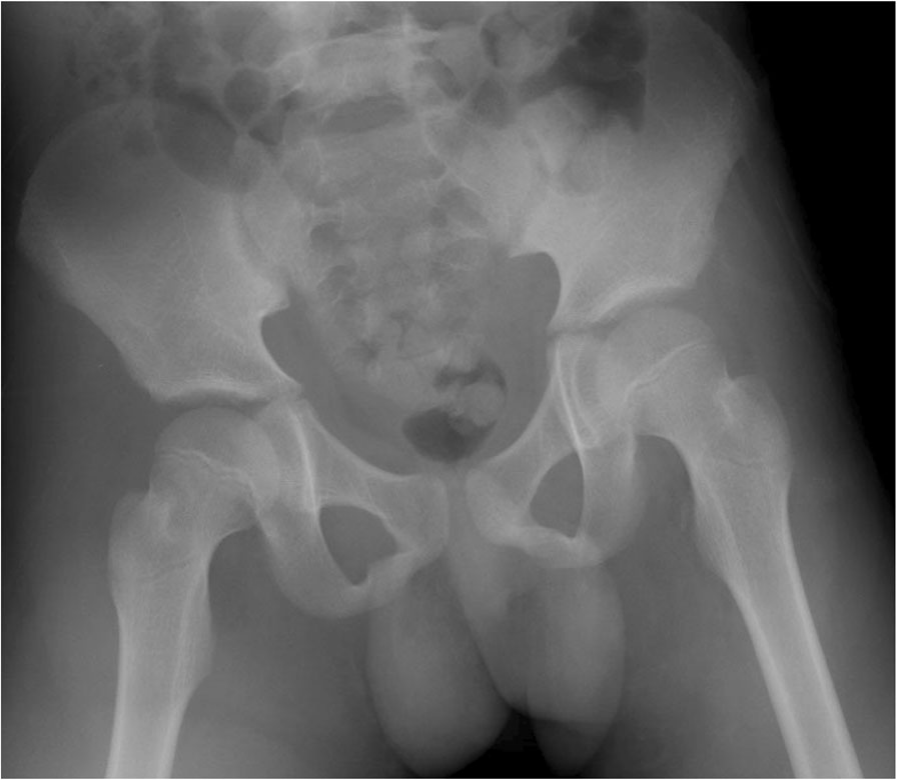
Postoperative anteroposterior radiograph showing concentric reduction of the right femoral head

The patient was sent to the operating room within 2 hours after admission. Soft tissue injury was found in the ligamentum teres, gluteus medius, gluteus minimus, iliopsoas, quadratus femoris, short external rotators, and capsule with avulsion fractures of the greater and lesser trochanters, indicating complete disruption of artery of ligamentum teres (Fig. 1c). Reduction of the hip joint was performed after thorough irrigation and debridement of the wound. Postoperative radiographs showed a concentrically reduced femoral head (Fig. 2).

**Fig. 3:**
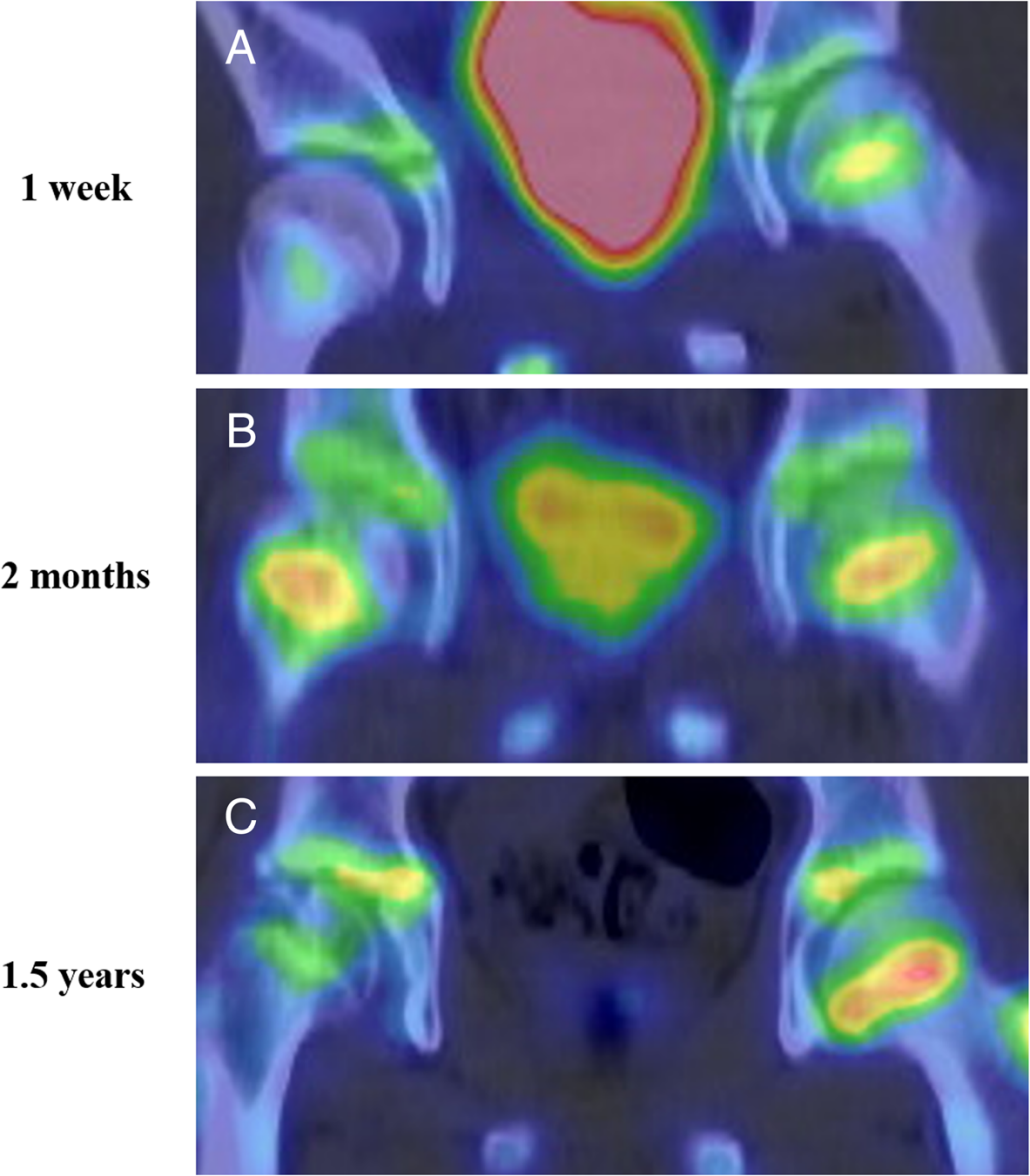
**a** Bone scintigraphy at 1 week after injury shows less accumulation on the metaphysis of the injured femoral head than on the contralateral side. **b** Bone scintigraphy at 2 months after injury shows increased accumulation over the epiphyseal plate. **c** Bone scintigraphy at 1.5 years after injury shows decreased accumulation over the epiphyseal plate, indicating early physeal arrest

**Fig. 4:**
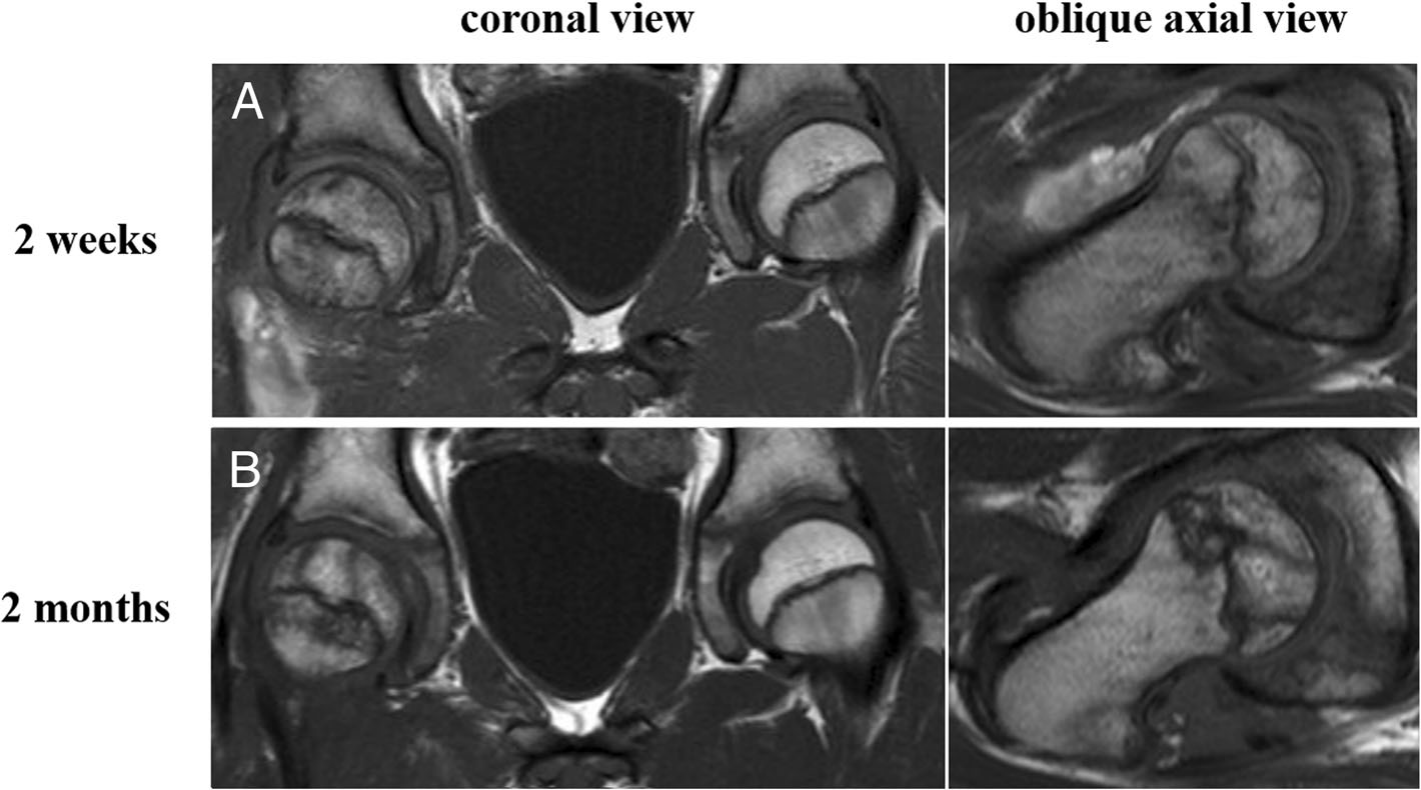
Coronal and oblique axial T1-weighted images at 2 weeks (**a**) after injury show diffuse area with low signal intensity in the proximal femur suggesting ischemia. Coronal and oblique axial T1-weighted images at 2 months (**b**) after injury show two bands with low signal intensity in the epiphysis of the femoral head, suggesting osteonecrosis

**Fig. 5:**
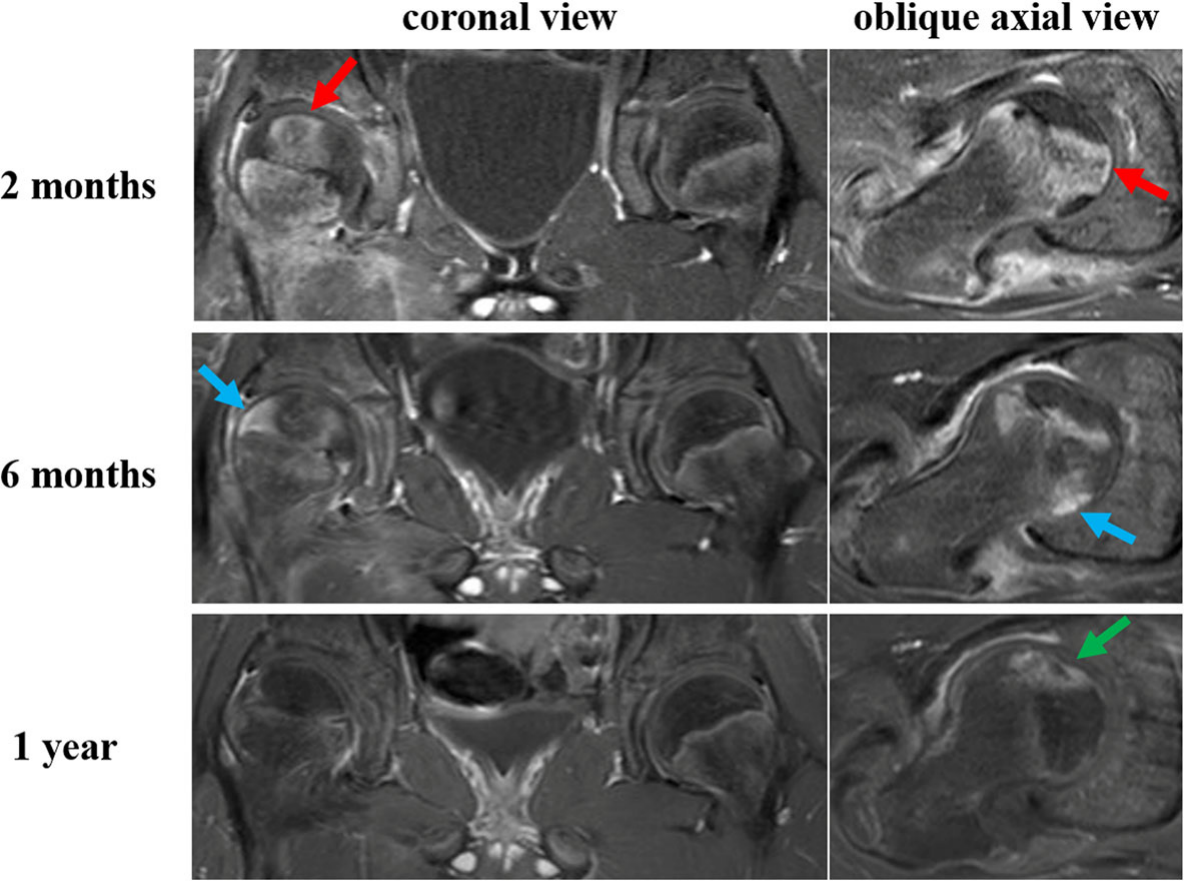
Coronal and oblique axial serial gadolinium-enhanced magnetic resonance images (MRIs) obtained at 2 months, 6 months, and 1 year. MRI at 2 months shows gadolinium enhancement in the central region (*red arrows*) and nonenhancement in the peripheral region of the femoral capital epiphysis. MRI at 6 months shows gadolinium enhancement spreading from the center toward the lateral and posterior regions of the femoral head (*blue arrows*). MRI at 1 year shows femoral head intensity equivalent to that on the contralateral side except for anterior region with slight collapse of articular surface of the femoral head (*green arrow*)

**Fig. 6:**
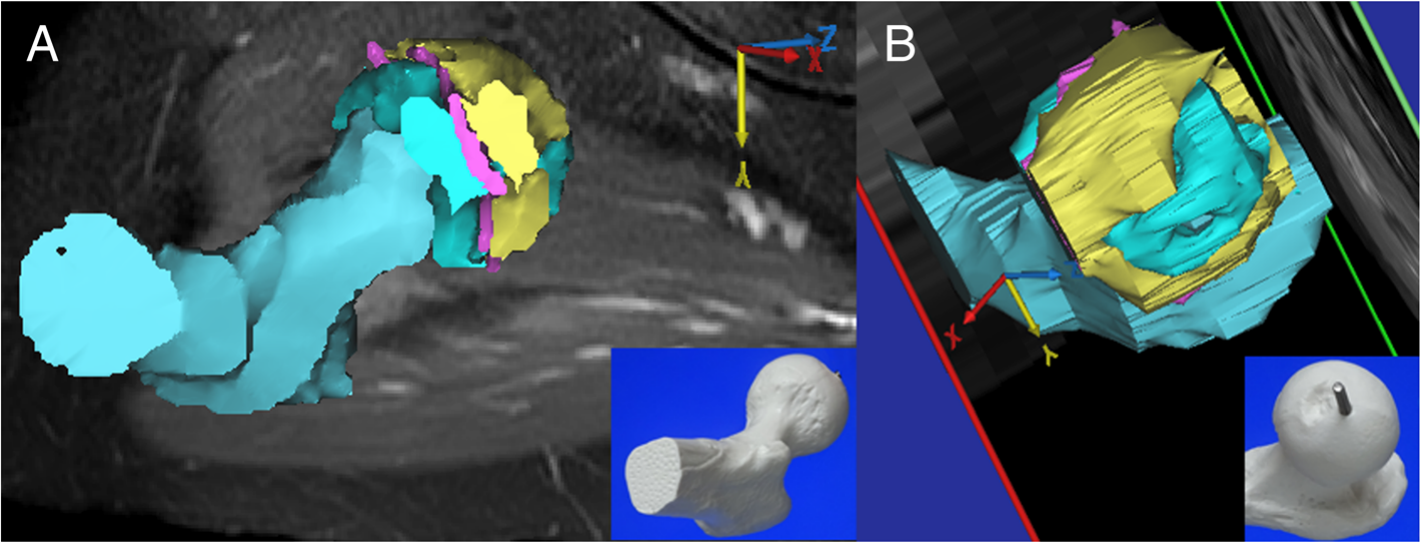
Three-dimensional proximal femoral models reconstructed from the gadolinium-enhanced magnetic resonance images at 2 months after injury. Blue, yellow, and purple structures represent the intact region, ring-shaped necrotic lesion, and epiphyseal plate, respectively. Anatomical models of the proximal femur at the lower right (**a** and **b**) represent the viewing direction

Bone scintigraphy (Symbia T6; Siemens Healthcare, Erlangen, Germany) at 1 week after injury, and immediate surgical reduction demonstrated less accumulation on the metaphysis of the injured femoral head than on the contralateral side, suggesting the existence of reduced bone turnover (Fig. 3a). T1-weighted magnetic resonance (MR) (ACHIEVA 1.5 T; Philips Healthcare, Amsterdam, the Netherlands) images (repetition time/echo time = 500 ms/ 18 ms, field of view = 320 mm, thickness = 5.0 mm) at 2 weeks after injury showed a diffuse area of low signal intensity on the proximal part of the femur compared with the contralateral side (Fig. 4a). Bone scintigraphy at 2 months showed increased accumulation over the epiphyseal plate that was similar to that on the contralateral side (Fig. 3b). T1-weighted MR images at 2 months after injury showed two bands with low signal intensity (bandlike pattern) [16] on both coronal and oblique axial views (Fig. 4b) and gadolinium enhancement in the area between the two low bands, suggesting the partial disruption of branches of superior and inferior retinacular arteries [17, 18] and existence of partial necrosis [19, 20] of the femoral capital epiphysis (Fig. 5). Three-dimensional proximal femur models including the epiphyseal plate, intact region, and necrotic lesion were reconstructed using Mimics software (Materialise NV, Leuven, Belgium) [21] and demonstrated a ring-shaped necrotic lesion adjacent to the growth plate (Fig. 6). Serial gadolinium-enhanced MRI at 6 months and 1 year showed a gradually enhanced area spreading from the center of the epiphysis toward the periphery, which indicated revascularization of the necrotic lesion with slight collapse of anterior articular surface of the femoral head (Fig. 5). Bone scintigraphy at 1.5 years showed decreased accumulation over the epiphyseal plate and accompanying premature physeal closure (Fig. 3c).

**Fig. 7:**
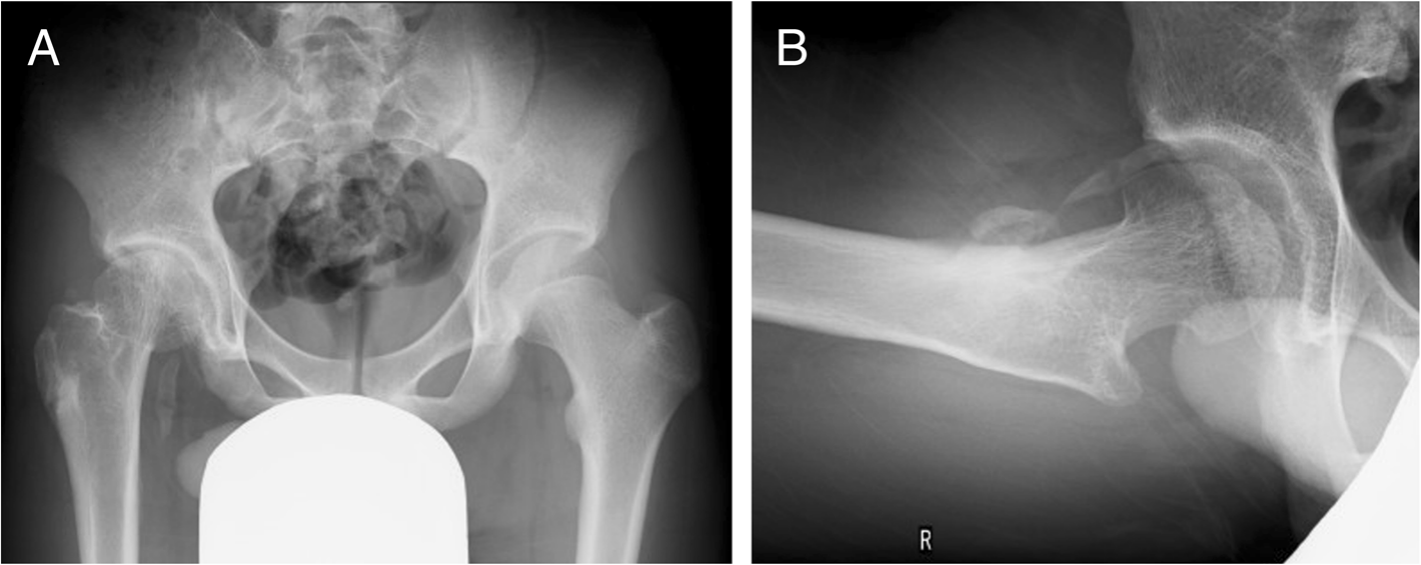
Anteroposterior (**a**) and lateral (**b**) radiographs at 4 years after injury show some resultant deformity of the femoral head, shortening of the femoral neck, and periarticular ossification without signs of secondary osteoarthritic change

Conservative non-weight-bearing treatment was applied on the affected right hip using a brace [22, 23] for 1.5 years after injury. Radiographs at 4 years after injury showed some resultant deformity of the femoral head and shortening of the femoral neck without narrowing of the joint space (Fig. 7). Although growth arrest resulted in a limb length discrepancy of 2 cm, the patient did not have any hip joint dysfunction or any signs of early osteoarthritis; he had a Harris Hip Scale score [24] of 100 points at 4.5 years after injury. He could return to light sports activity without any pain or limited range of motion.

## Discussion

Traumatic open anterior dislocation of the hip in children is associated with serious complications and could have a very poor prognosis [1, 5, 9, 10, 25–29]. In our patient’s case, bone scintigraphy after prompt reduction indicated reduced bone turnover on the femoral head due to the severity of the initial trauma [29, 30]. T1-weighted MR images at 2 months after injury showed two bands with low signal intensity in the femoral capital epiphysis, suggesting the existence of osteonecrosis. Gadolinium-enhanced MR images at 2 months showed nonenhanced area in the peripheral region of the femoral capital epiphysis, indicating disruption of artery of the ligamentum teres and branches of superior retinacular artery [17, 18].

Bohr *et al.* [31] examined the vascular supply to the femoral head following dislocation of the hip joint in newborn rabbits and showed that revascularization takes place through vessels from the trochanteric region and by vessels perforating the epiphyseal plate from the metaphyseal side. Conway *et al*. [32] reported that revascularization of the necrotic proximal femoral epiphysis in Legg-Calvé-Perthes disease can occur by rapid recanalization of existing vessels or by prolonged neovascularization through the development of new vessels. Kim *et al.* observed revascularization occurring from the periphery to the central region [15], which is different from our patient’s case. However, no previous report demonstrated the revascularization process of osteonecrosis after traumatic hip dislocation. In this case, serial MR images showed the gadolinium-enhanced region gradually spreading from the center of the epiphysis toward the peripheral region, indicating appreciable revascularization.

Weight-bearing within 6 weeks without accurately assessing either the necrotic area or revascularization might lead to premature breakdown of the hip joint in children with ischemic necrosis after traumatic hip dislocation [5, 9, 10, 14, 33]. Collapse of the necrotic femoral head could occur with earlier weight-bearing during the revascularization process due to the weakness of the affected head resulting from the mechanical stress [34]. Kim *et al*. reported that joint loading due to muscle contractions can also contribute to the development of the femoral head deformity, but local non-weight-bearing could provide significantly better preservation of the round shape of the femoral head [14, 15]. They reported the gradual revascularization process of the femoral epiphysis in Legg-Calvé-Perthes disease using serial perfusion MRI and recommended that the non-weight-bearing period be based on a certain level of normalization of the signal intensity in the revascularized area due to reossification. In our patient’s case, repeated evaluation of the necrotic area and revascularization using gadolinium-enhanced MRI helped in the careful planning of appropriate time for weight-bearing. Although osteonecrosis in the anterior region caused slight collapse of articular surface of the femoral head, conservative treatment with non-weight-bearing for 1.5 years for the affected hip could achieve favorable outcomes within 4.5 years after injury.

Growth disturbances causing damage to the physis in the peripheral region including the perichondral structures, which play an essential role in the growth of the width and length [35], result in deformity of the proximal femur with leg-length discrepancy [12]. Although long-term follow-up is necessary for early detection of hip arthritis, our patient returned to performing his normal activities without any limitation at 4.5 years after injury.

## Conclusions

We confirmed the revascularization process of the necrotic lesion in the femoral capital epiphysis after traumatic open anterior dislocation of the hip joint in an 11-year-old boy using serial gadolinium-enhanced MRI. Although he had deformity of the proximal femur with leg-length discrepancy, non-weight-bearing treatment using a brace for 1.5 years achieved a favorable outcome without any hip joint dysfunction, pain, or sign of secondary osteoarthritic change within 4.5 years after injury.

## Data Availability

Medical imaging data will not be shared, because it is not fully anonymous.

## Abbreviations

MR: Magnetic resonance
MRI: Magnetic resonance imaging
